# Supplementary material for: Analysis of immune, microbiota and metabolome maturation in infants in a clinical trial of Lactobacillus paracasei CBA L74-fermented formula
Source: Nat Commun. 2020 Jun 1;11:2703. doi: 10.1038/s41467-020-16582-1 (PMC7264213; doi:10.1038/s41467-020-16582-1)
Supplement: Supplementary file 1 — Supplementary Information [file 41467_2020_16582_MOESM1_ESM.pdf]

Supplementary Information

Analysis of immune, microbiota and metabolome maturation in infants in a clinical trial of *Lactobacillus paracasei* CBA L74-fermented formula

Roggero et al.

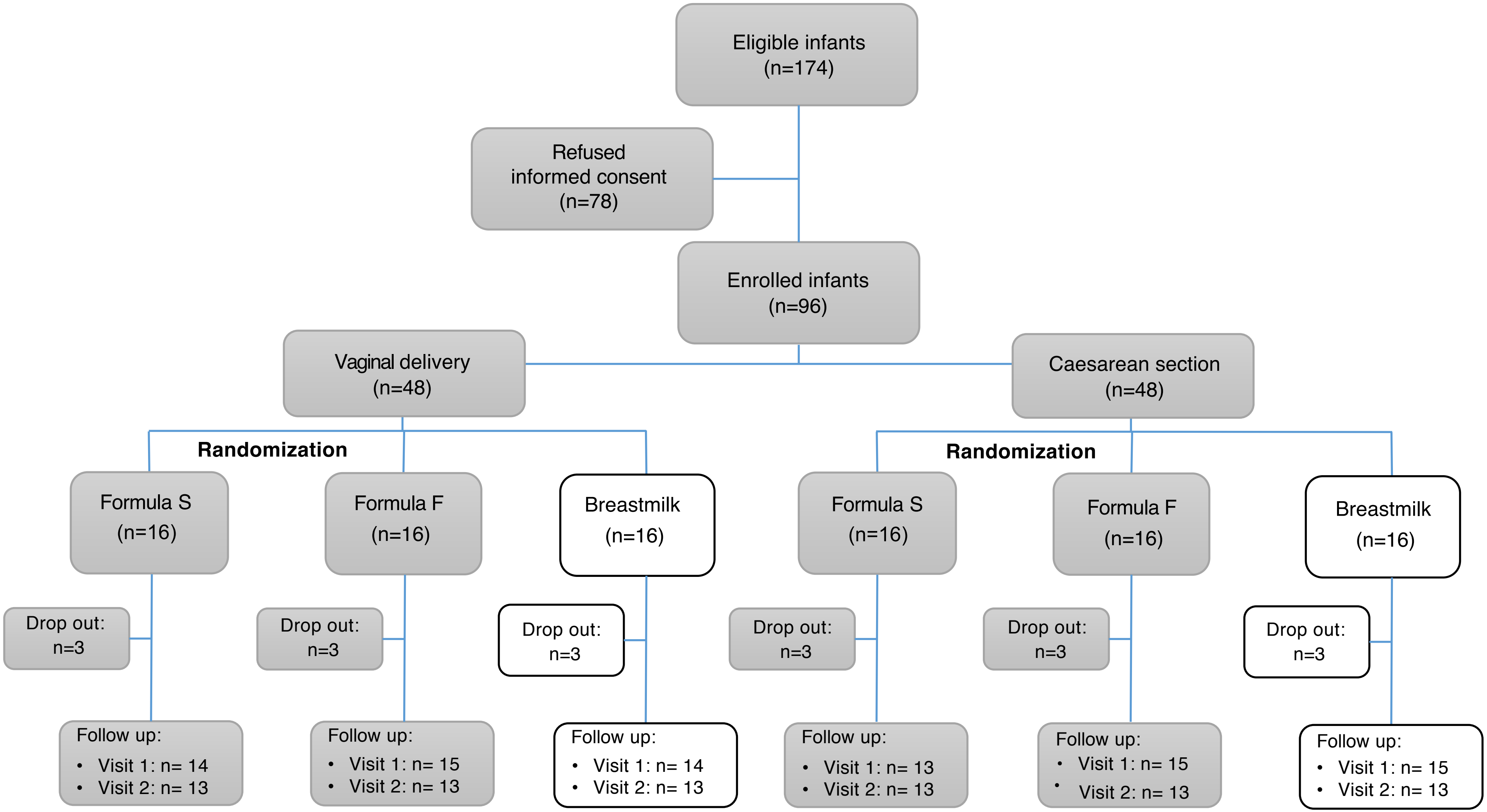

Supplementary Figure 1. Diagram showing the enrolment and follow up of participants.

Supplementary Table 1 Basic characteristics at birth of infants included according to the mode of delivery and the type of feeding

	Vaginal Delivery			Caesarean section		
Dietary regimen	Formula S (n=13)	Formula F (n=13)	Breastmilk (n=13)	Formula S (n=13)	Formula F (n=13)	Breastmilk (n=13)
Weight (g)	3264 ± 340	3253 ± 455	3314 ± 363	3123 ± 305	3196 ± 347	3302 ± 440
Length (cm)	49.9 ± 1.5	49.3 ± 1.3	49.9 ± 1.9	49.0 ± 1.6	49.3 ± 1.9	49.9 ± 1.6
Head circumference (cm)	33.8 ± 0.8	33.8 ± 1.1	34.3 ± 1.0	34.1 ± 1.0	34.5 ± 1.0	34.0 ± 1.3
Males n (%)	6 (50.0)	2 (15.3)	5 (38.5)	5 (41.6)	4 (33.3)	4 (33.3)

No differences in anthropometric measurements were found among groups at birth. Analysis of variance was used to assess differences in continuous variables and the χ^2 test for comparisons discrete variables between groups.

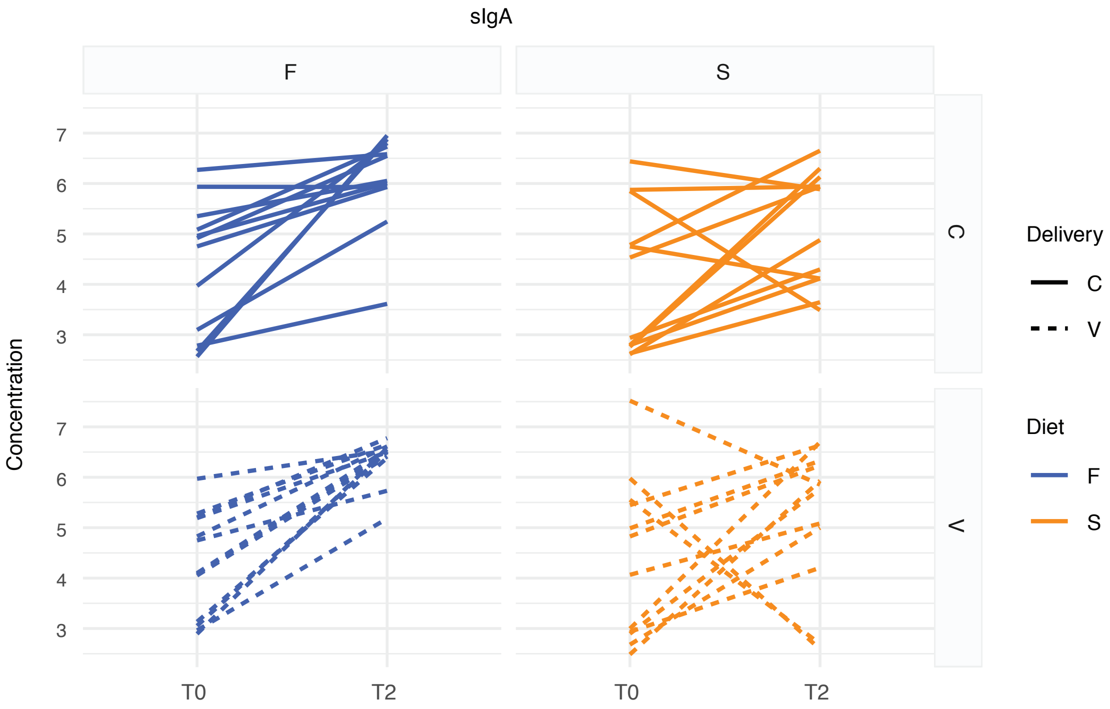

Supplementary Figure 2. Fermented formula drives more sIgA than Standard formula. sIgA levels according to mode of delivery and type of feeding were determined in fecal samples and are represented at T0 and T2. Each individual infant is represented by a line (solid lines caesarean delivery, C; dotted lines vaginal delivery, V). F and S fed infants show significantly different trajectories of IgAs levels between T0 and T2, with F fed infants displaying higher levels of IgAs and all increasing from T0 to T2 (ANOVA $P = 0.010$). Simple effects T0-T2: F diet (Caesarean $P = 0.0018$, Vaginal $P = 3.38E-05$), S diet (Caesarean $P = 0.779$, Vaginal $P = 0.902$). Significance determined accounting for individual baselines with a linear mixed model, ANOVA to test the interaction between groups and time and Tukey post-hoc test. Data are shown in log_2 scale. Source data are provided as a Source Data file.

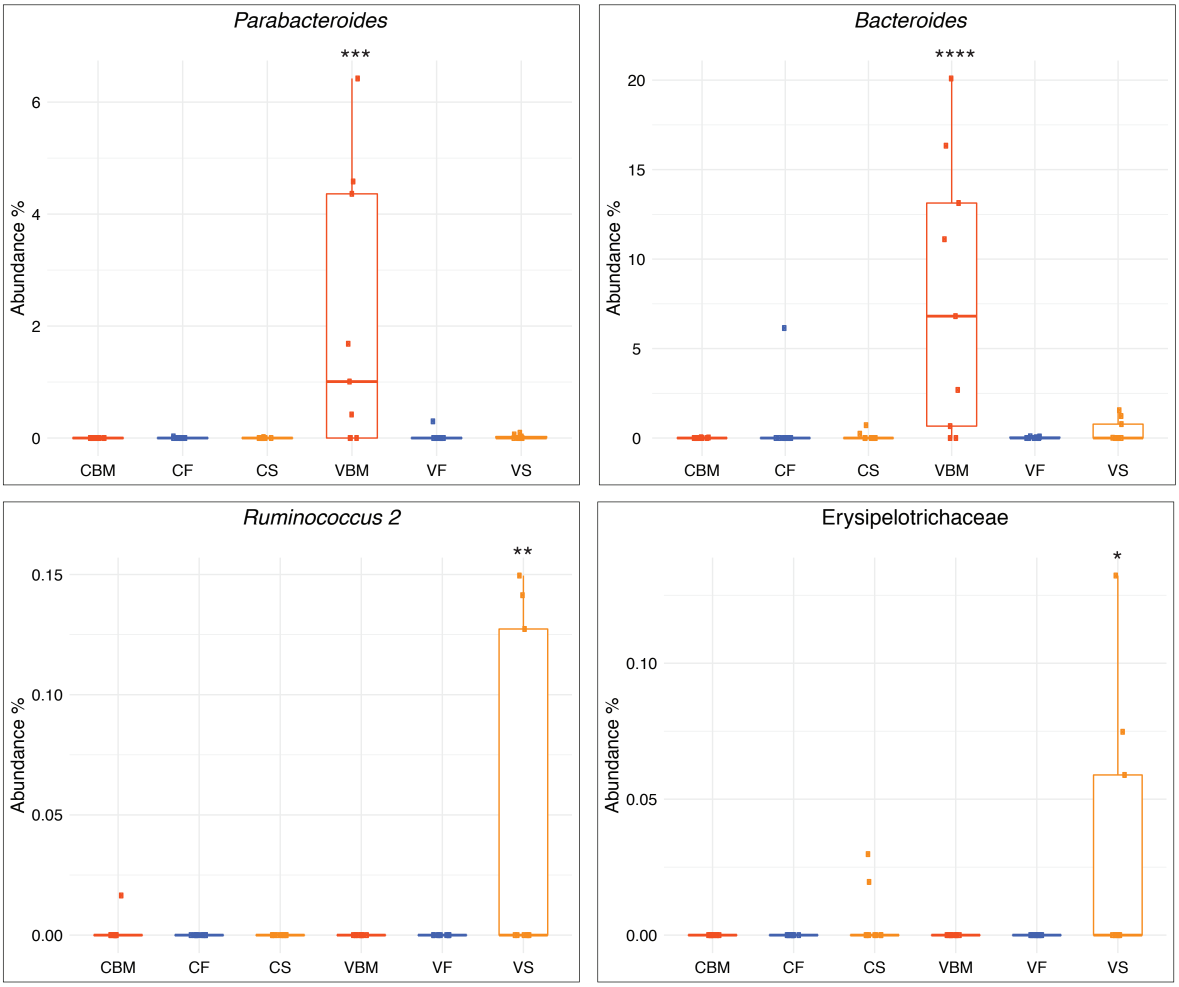

Supplementary Figure 3. Bacterial genera enriched in the different diet groups by mode of delivery at visit 2. Box plots showing differential abundance of *Parabacteroides, Bacteroides, Ruminococcus 2* and Erysipelotrichaceae. Box plots show the interquartile range (IQR), the horizontal lines show the median values and the whiskers extends from the hinge no further than 1.5 * IQR. Each dot represents individual newborns. CBM: caesarean delivery breastmilk feeding; CF: caesarean delivery fermented formula feeding; CS: caesarean delivery standard formula feeding; VBM: vaginal delivery breastmilk feeding; VF: vaginal delivery fermented formula feeding; VS: vaginal delivery standard formula feeding. n = 9 infants per group. Significance determined by ANOVA. *$P < 0.05$, **$P < 0.01$, ***$P < 0.001$, ****$P < 0.0001$. Significant Tukey post-hoc comparisons are shown in Supplementary Table 2. Source data are provided as a Source Data file.

Supplementary Table 2 List of significant bacterial genera showing different abundance across the diet groups by mode of delivery at T2

Genus	ANOVA *p*-value[a]	TukeyHSD post-hoc test[b]
Bacteroides	8.80856E-06	VBM-CBM *P* = 6.4E-05, VBM-CF *P* = 0.0002, VBM-CS *P* = 8.0E-05, VF-VBM *P* = 6.76-05, VS-VBM *P* = 0.0001
Parabacteroides	0.000138412	VBM-CBM *P* = 0.0008, VBM-CF *P* = 0.0008, VBM-CS *P* = 0.0008, VF-VBM *P* = 0.0010, VS-VBM *P* = 0.0009
Ruminococcus 2	0.004829657	VS-CBM *P* = 0.0208, VS-CF *P* = 0.0144, VS-CS *P* = 0.0144, VS-VBM *P* = 0.0144, VS-VF *P* = 0.0144
Erysipelotrichaceae	0.01761655	VS-CBM *P* = 0.0364, VS-CF *P* = 0.0364, VS-VBM *P* = 0.0364, VS-VF *P* = 0.0364

[a,b]Significance determined by ANOVA and Tukey post-hoc test. n = 9 infants per delivery-diet group.
[b]Comparisons between groups with significant post-hoc analysis ($P < 0.05$).

Supplementary Table 3 List of significant bacterial genera showing different abundance across the diet groups at T2

Genus	ANOVA *p*-value[a]	TukeyHSD post-hoc[b]
Bacteroides	0.008937096	F-BM *P* = 0.022, S-BM *P* = 0.018
Parabacteroides	0.013187348	F-BM *P* = 0.028, S-BM *P* = 0.027
Clostridium innocuum group	0.029880051	S-F *P* = 0.035, S-BM *P* =0.090
Erysipelotrichaceae	0.020307833	S-BM *P* = 0.039, S-F *P* = 0.039
Veillonella	0.008703064	F-BM *P* = 0.025, S-BM *P* = 0.015
Ruminococcus 2	0.047468236	S-BM *P* = 0.088, S-F *P* = 0.072

[a,b]Significance determined by ANOVA and Tukey post-hoc test. n = 18 infants per diet group.
[b]Comparisons between groups with significant post-hoc analysis ($P < 0.05$).

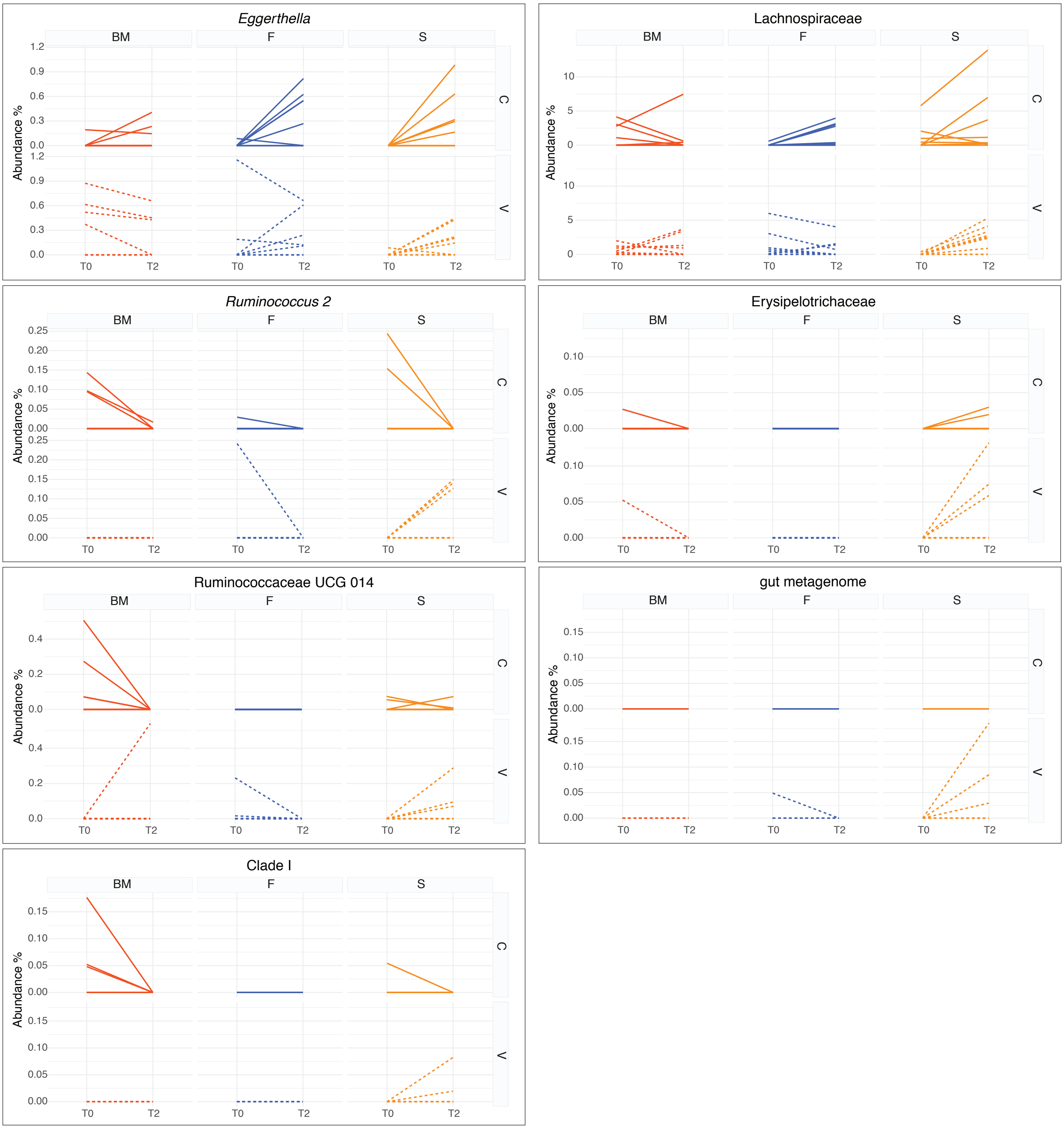

Supplementary Figure 4. Bacterial genera trends in the different diet groups by mode of delivery for each individual infant between T0 and T2. Interaction plots of abundance of statistically different genera (T0-T2) according to diets and delivery mode. Each line represents individual newborns (solid lines caesarean delivery, C; dotted lines vaginal delivery, V). Significance determined accounting for individual baselines with a linear mixed model and ANOVA to test the interaction between groups and time. ANOVA *P* value: *Eggerthella P* = 0.011, Lachnospiraceae *P* = 0.028, *Ruminococcus 2 P* = 0.019, Erysipelotrichaceae *P* = 0.005, Ruminococcaceae UCG 014 *P* = 0.029, gut metagenome *P* = 0.020, Clade I *P* = 0.027. BM: breastmilk feeding; F: fermented formula feeding; S: standard formula feeding. Source data are provided as a Source Data file.

Similar trend between F and S formulas

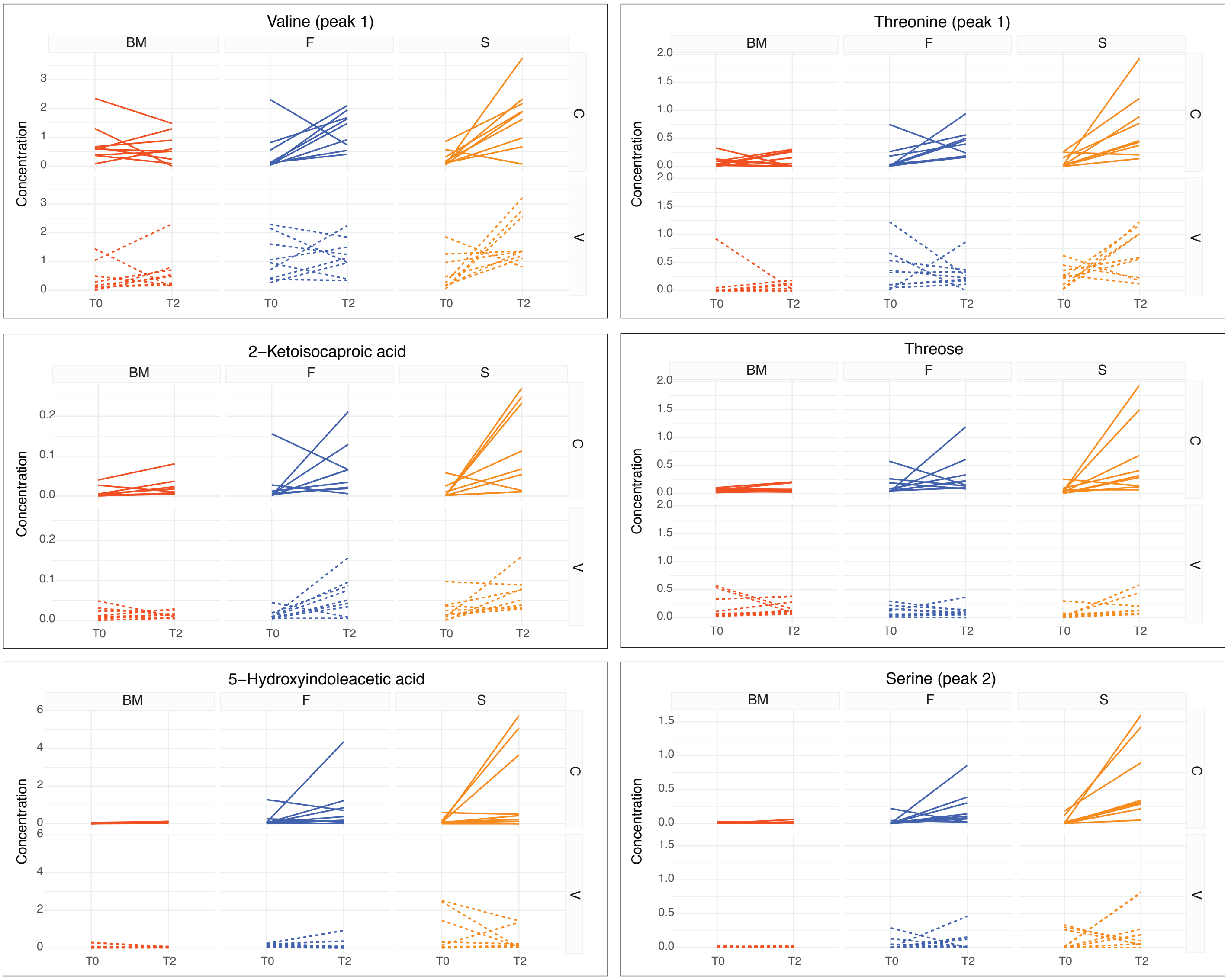

Similar trend of F formula versus reference group

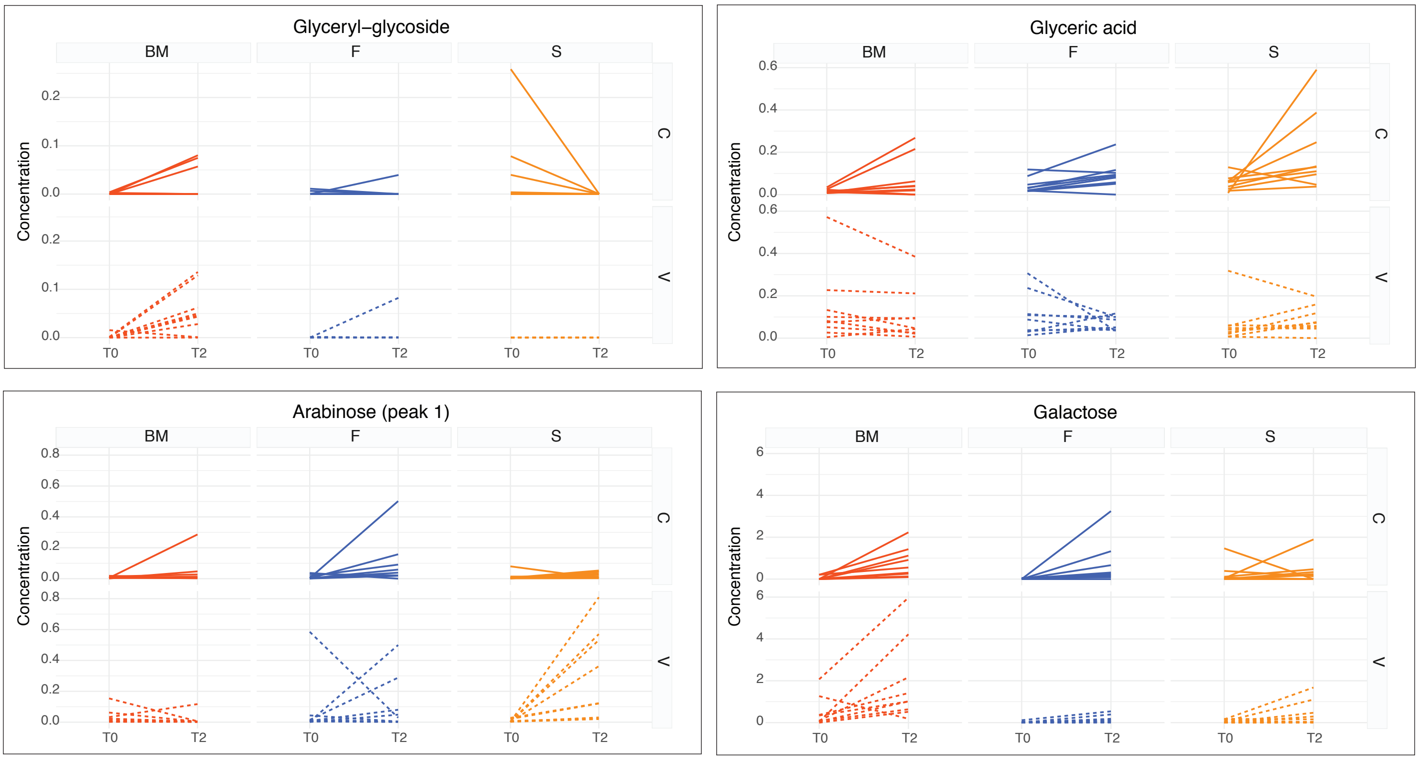

Supplementary Fig. 5 ->

Reduced or absent in formula fed versus the reference group

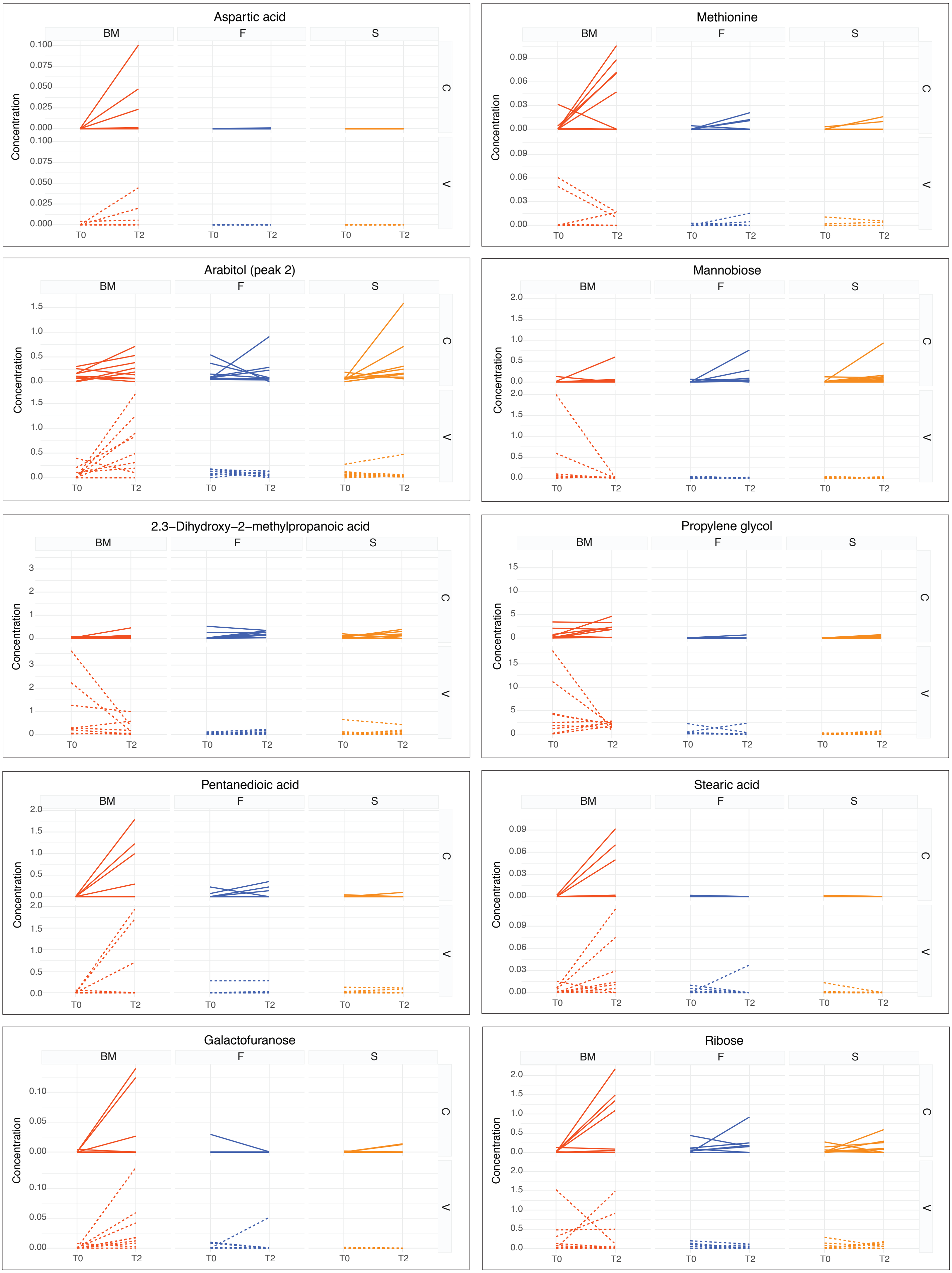

Supplementary Fig. 5 ->

<-

Supplementary Figure 5. Metabolite trends in the different diet groups by mode of delivery for each individual infant between T0 and T2. Interaction plots of abundance of statistically different metabolites (T0-T2) according to diets and delivery mode. Each line represents individual newborns (solid lines caesarean delivery, C; dotted lines vaginal delivery, V). Significance determined accounting for individual baselines with a linear mixed model and ANOVA to test the interaction between groups and time. ANOVA *P* value: Valine peak 1 *P* = 0.0012, Threonine peak 1 *P* = 0.0005, 2-Ketoisocaproic acid *P* = 0.0107, Threose *P* = 0.00270, 5-Hydroxyindoleacetic acid *P* = 0.0068, Serine peak 2 *P* = 0.0005, Glyceryl glycoside *P* = 0.0004, Glyceric acid *P* = 0.0041, Arabinose peak 1 *P* = 0.0040, Galactose *P* = 0.0404, Aspartic acid *P* = 0.0322, Methionine *P* = 0.0001, Arabitol peak 2 *P* = 0.0024, Mannobiose *P* = 0.0238, 2-3-Dihydroxy-2-methylpropanoic acid *P* = 0.0168, Propylene glycol *P* = 0.0276, Pentanedioic acid *P* = 0.0137, Stearic acid *P* = 0.0158, Galactofuranose *P* = 0.0172, Ribose *P* = 0.0155. BM: breastmilk feeding; F: fermented formula feeding; S: standard formula feeding. Source data are provided as a Source Data file.

Supplementary Table 4 Primers used in this study

Primer Name	Adapter Sequence	Key	Tag barcode	GAT	Primer Sequence (5'–3')	Ref.
Probio_Uni	CCATCTCATCCCTGCGTGTCTCCGAC	TCAG	TTGGAGTGTC	GAT	CCTACGGGRSGCAGCAG	55
Probio_Rev	CCTCTCTATGGGCAGTCGGTGAT				ATTACCGCGGCTGCT	55
