# Supplementary material for: Analysis of immune, microbiota and metabolome maturation in infants in a clinical trial of Lactobacillus paracasei CBA L74-fermented formula
Source: Nat Commun. 2020 Jun 1;11:2703. doi: 10.1038/s41467-020-16582-1 (PMC7264213; doi:10.1038/s41467-020-16582-1)
Supplement: Supplementary file 3 — Description of Additional Supplementary Files [file 41467_2020_16582_MOESM3_ESM.pdf]

Description of Additional Supplementary Files

File Name: Supplementary Data 1

Description: Study Protocol Document
