# Supplementary material for: Analysis of immune, microbiota and metabolome maturation in infants in a clinical trial of Lactobacillus paracasei CBA L74-fermented formula
Source: Nat Commun. 2020 Jun 1;11:2703. doi: 10.1038/s41467-020-16582-1 (PMC7264213; doi:10.1038/s41467-020-16582-1)
Supplement: Supplementary file 4 — Supplementary Data 1 [file 41467_2020_16582_MOESM4_ESM.pdf]

Dipartimento Donna-Bambino-Neonato
UOC Neonatologia e Terapia Intensiva Neonatale - Direttore: **Prof. Fabio Mosca**
Tel. 02. 5503.2907
mail: neonatologia@policlinico.mi.it

Modification of immunity in the newborn at term in relation to the type of breastfeeding and mode of delivery

Study code: FERCT15

FERCT15_Protocollo_v2_10.06.15

INDEX

1. Investigators and administrative structure of the study

2. Introduction

3. Objectives of the study

3.1. Primary objectives

3.2. Secondary objectives

4. Study design

5. Duration of the study

6. Selection of the study population

6.1. Inclusion criteria

6.2. Exclusion criteria

6.3. Exclusion of infants from the study and end of study criteria

7. Study procedure

7.1. Enrolment

7.2. Assignment to treatment

7.3. Evaluations performed during the study

8. Sample collection and measurement methods

8.1. Anthropometry

8.2. Body composition

8.3. Stool collection

8.4. Evaluation of Tolerance

8.5. CRF patient

8.6. Concomitant therapies

9. Product description

9.1. Composition

9.2. Dosages

9.3. Quality check

9.4. Packaging and labelling

9.5. Product distribution and storage

10. Adverse events

10.1 Reporting of serious adverse events

10.2 Follow-up of adverse events

10.3 Post-study serious adverse events

10.4 Definition of the relationship

10.5 Responsibility for reporting serious adverse events

11. Statistical analysis

11.1 Calculation of the sample size

11.2 Criteria for the evaluation of variables

12. Legal and ethical requirements

12.1 Legality of the study

12.2 Protection of patient data privacy

12.3 Informed consent

12.4 Paediatrician Care Information

12.5 Approval by the ethics committee

12.6 Quality Assurance and Quality Control

13. Responsibility

13.1 Responsibilities of the investigators

13.2 Responsibilities of the sponsor

14. Documentation and access to data

14.1 Documentation of the participation of the subjects in the study

14.2 Documentation of essential documents

14.3 Data protection

14.4 Documents of study participants

15. Administrative procedures

15.1 Amendments to the protocol

15.2 Disclosure of information and results

15.3 Premature termination of the study

15.4 Insurance for infants

15.5 Registration of the protocol

16. Bibliography

1. Investigators and administrative structure of the study

The trial will take place at the Neonatology and Neonatal Intensive Care Unit, Mangiagalli Clinic, IRCCS Ca 'Granda Foundation, Ospedale Maggiore Policlinico.

Investigators should be familiar with the requirements and obligations of Good Clinical Practice (GCP). Investigators and center staff will receive a specific training study during the week preceding the start of recruitment.

Center for the study protocol

Mangiagalli Clinic

IRCCS Foundation Ca 'Granda, Ospedale Maggiore Policlinico

University of Milan

Via Commenda 12

20122 Milan (MI)

Responsible Investigator: Prof Fabio Mosca

Director of the U.O. of Neonatology and Neonatal Intensive Care Department of Clinical and Community Sciences, University of Milan IRCCS Foundation Cà Granda Ospedale Maggiore Policlinico Via Della Commenda 12 - 20122 Milan Secretary: tel. 02 / 5503.2907- fax 02 / 5503.2429 Email: fabio.mosca@mangiagalli.it Email: fabio.mosca@unimi.it

Principal Investigator:

Dr. Paola Roggero Nutrition Center at neonatal start, Neonatology and Neonatal Intensive Care Unit, Mangiagalli Clinic IRCCS Foundation «Ca'Granda» Ospedale Maggiore Policlinico Via Commenda 12, 20123 Milan, Italy tel.02 / 5503.2483 - fax 02 / 5503.2436 Email: paola.roggero@mangiagalli.it

Sponsor Company: Heinz Italia S.p.A., Via San Bovio n. 1/3 20090 Segrate (MI) Tel: 00 39 02 5256 1

2. Introduction

The microbiota plays an important role in modulating the development of the immune system, making critical the interrelation that is created between nutrition, microbiota and immune cells relative to long-term health outcomes. The factors that contribute to the development of the microbiota are numerous. Among them, nutrition, in particular breastfeeding, and the type of birth have been the object of greater study in recent years. Exclusive breastfeeding, recommended in accordance with the World Health Organization, promotes the development of a microbiota in which bifidobacteria are particularly represented and promotes protection against potential infectious agents and the correct development of the immune system. Formulas which represent the substitutes for breastmilk, when this is not available or there are contraindications to breastfeeding, have different characteristics in terms of composition and consequently of function. Caesarean section alters the development of the bacterial flora, hindering the exposure of the newborn to the microbiota of maternal origin.

In recent years, attempts have been made to improve the biological effects of formulas. Functional food derived from the fermentation of cow's milk with probiotic strains has been proposed for the prevention of children infectious diseases. Several products have been investigated, with sometimes

conflicting results. Diversity in experimental study designs, in study populations, and in bacterial strains used in the preparation of fermented products are probably responsible for these discrepancies. Recent scientific evidence has shown the beneficial effect of formulated milk added with fermented products without live bacteria in healthy infants. The benefits can be ascribed to the bacterial components that remain in the final product (for example DNA, cell wall, etc.) and to the factors produced during fermentation (short chain fatty acids, bacterial proteins, etc.). The main effects of these bacterial components concern the stimulation of the lymphoid tissue associated with the gastrointestinal tract through interaction with immune cells via Toll-like receptors.

It has been documented that fermented infant formulas increase the amount of bifidobacteria and promote the development of specific IgA secretions, polio-viruses, in response to Pentacoq vaccination, compared to infants fed with standard formula. Another randomized controlled trial, conducted on two groups of full-term infants fed with milk formula fermented or with standard formula and compared with a group of infants breastfed, has shown that infants fed with fermented formula compared to infants fed with standard formula had thymus sizes and fecal pH values similar to those found in infants fed breast milk. In another study, conducted on 971 infants aged between 4 and 6 months, the beneficial effects of a long-term administration of a milk added with a fermented product were reported with respect to a group of infants fed with the standard formula on the severity of the diarrhea. The use of formulas added with fermented products is safe since it allows a growth not inferior to children fed with standard formula. Based on the available scientific evidence, the Nutrition Commission of the The European Society of Gastroenterology, Hepatology and Pediatric Nutrition (ESPGHAN) therefore stated that fermented formulated milks are safe and potentially effective in determining a prebiotic effect and reducing the severity of infectious diarrhea episodes. The efficacy of using fermented milk with the probiotic Lactobacillus paracasei CBA L74 was recently evaluated in the decrease of the incidence of infectious diseases during the winter period in 259 children (12-48 months) who frequented the community. The study participants were randomized to receive fermented milk or a placebo (maltodextrin) for a period of three months. Children fed with fermented milk developed infections of the upper respiratory tract and the gastrointestinal tract in a percentage of cases significantly lower than those taking placebo

(decrease in the percentage 48.2% vs 70.5 p <0.001 and 13.1% vs 31.1%, p <0.0001, respectively). There was also a significant increase in alpha and beta defensins, LL37 and secretory IgA in children taking fermented milk compared to the placebo group.

Fermented formulated milk

The functional food object of this study, that is formula milk for newborns fermented with the probiotic Lactobacillus paracasei CBA L74, falls into the category of "postbiotics". At the end of the fermentation process the probiotic is inactivated through a heat treatment. The finished product, therefore, does not contain live bacteria. Pre-clinical phase studies have shown an anti-inflammatory property of fermented milk with Lactobacillus paracasei CBA-L74 in terms of stimulation of cytokine IL-10 production and reduction of synthesis IL-12, also in response to stimulation with Salmonella typhimurium. The data were obtained in in vitro studies on dendritic cells and ex vivo on intestinal biopsies as well as in tests on healthy mice and on a mouse model of experimental colitis. The bacterium used in the fermentation process was deposited by the Sponsor in the Belgian collection BCCM / LMG with the deposit code LMG S-24480. This bacterium is part of the Lactobacillus paracasei species and is included in the list of "Qualified Presumption of Safety (QPS) microorganisms" compiled by the Panel on Biological Hazards of the European Food Safety Authority. The bacterium was also tested for the absence of antibiotic resistance genes in accordance with the guidelines published by the "Panel on Additives and Products or Substancesused in Animal Feed" by EFSA. The bacterium was also genetically characterized through the identification of its molecular profile by Rep-PCR technique (RepetitiveExtragenicPalindromic - PCR). The bacterium was selected for its ability to grow on milk. The bacterial load at the end of the fermentation process and before the spraying that inactivates the cells is about 109 CFU / g of product.

3. Purpose of the study:

Primary:

To evaluate whether feeding with milk formulated for infants fermented with Lactobacillus paracaei CBA L74 results in an increase in anti-microbial peptides such as catelecidins, alpha and beta defensins and secretory IgA compared to feeding with the standard formula (Plasmon Primigiorni), with reference to milk breast.

Secondary:

- To evaluate the tolerance in the two groups of infants fed with the two formulas in the study, in reference to the group of infants fed with breast milk.

- To evaluate the modifications of the intestinal microbiota in the two groups of infants fed with the two formulas in the study, in reference to the group of infants fed with breast milk.

4. Study design

Double-blind randomized monocentric controlled study in parallel groups with reference group.

5. **Duration of the study**

The study will last for 36 months; a 30-month enrollment period and a 3-month intervention period are envisaged.

6. **Selection of the study population**

The Policlinico Maggiore Hospital is eligible to study at the IRCCS "Ca Granda" Foundation

6.1 Inclusion criteria

1. Healthy term newborns from normal pregnancy

2. Gestational age between 37 and 41 weeks

3. Appropriate birth weight between 10th and 90th centile World Health Organization (ANNEX 1)

4. Caucasian and non-Caucasian ethnicity

5. Absence of maternal milk

6. Contraindications to breastfeeding

6.2 Exclusion criteria

1. Newborns with birth weight <10 and> 90th centile World Health Organization

2. Congenital malformations, chromosomopathies, cardiopathies, gastrointestinal, respiratory, neurological and metabolic diseases

3. Perinatal infections

4. Positive familiarity for milk protein allergy

5. Infants whose parents expect a transfer within 3 months of birth

Reference group

Infants fed with exclusive breast milk for the first three months of life.

6.3 Exclusion of infants from the study and end of study criteria

In the case of infants who do not attend the study visits, every effort should be made to trace them, recall them and at least make sure of their state of health. All these attempts must be documented in the infant's data collection form and in the original documents.

The reasons for stopping the study will be classified as follows:

- Voluntary interruption
- Suspension decided by the investigator or by the trusted pediatrician
- Adverse event which in the opinion of the investigator requires suspension
- Suspension decided by the sponsor
- Infants lost at follow-up
- Major breach of the protocol
- Other reason (specify)

It is considered that an infant is assessable with respect to the primary and secondary objectives if the infants will make all the assessments up to the age of 3 months.

7. Study procedure

7.1 Enrollment

Newborns will be enrolled at birth after obtaining the informed consent of both parents.

At the time of enrollment, newborns who, due to the absence of breast milk, take artificial milk will be randomized to receive the formula in the study or the control formula until the third month of

age. The packs of the two formulas will be the same and identified with letters so as not to make the typology recognizable either to the experimenter or to the parents of the participants. The details of the two formulas in the study are described in ANNEX 4.

Enrollment will be carried out by promoting and supporting breastfeeding and, in the case of exclusive breastfeeding, infants will be included in the study as a reference group.

7.2 Assignment to treatment

The assignment of infants to one of the formulas will be double-blind (both the experimenter and the parents of the participants must not know which formula the child is taking), based on different randomization lists based on the methods of delivery (spontaneous birth / caesarean section), which will be provided by the Sponsor. On the same occasion, sealed copies of the randomization codes that will be kept in the investigator's archive will be delivered. In the event of a Serious Adverse Event (SAE) for which it is necessary to know the composition of the formula taken by the infant, the experimenter will be able to open the code in order to better manage the SAE. Each infant will be identified throughout the duration of the study by the initials (the first letter of the name followed by the first letter of the surname) and by an alphanumeric identification code, corresponding to the progressive alphanumeric code assigned at the time of recruitment by the investigator for consultation of the randomization list .

7.3 Evaluations performed during the study

During the study there are 3 study points: enrollment (within 7 days of life, V0), first (V1) and third (V2) month of life (Table 1).

Parameters controlled at V0

On the occasion of the first meeting with the parents / guardians the Investigator, after having assessed the respect of the inclusion / exclusion criteria for the newborn, will request to sign the informed consent form for both parents. Each newborn will be randomized into one of the two Study Groups and the parents of each newborn will be given the dietary product assigned for the total period of study and a diary for the collection of data relating to tolerance (ANNEX 2). Parents will be instructed by the Investigator on the use of the product and on data collection. On this occasion, a stool sample will be collected and the anthropometric parameters (weight, length and head circumference) and body composition (fat mass and lean mass) of the newborn will be evaluated.

Parameters checked at V1

A sample of stools from the newborn will be collected. Evaluation of the anthropometric parameters (weight, length and head circumference) of each newly enrolled infant will also be performed.

Parameters controlled at V2

A sample of stools from the newborn will be collected. Evaluation of the anthropometric parameters (weight, length and head circumference) and of body composition (fat mass and lean mass) of each enrolled newborn will also be performed. On this occasion, the clinical diary given to the parents at the time of birth will be withdrawn.

Table 1: V0: enrollment and visit within 7 days of life V1 and V2: visit at 1 and 3 months of age

**Procedures**	**V0**<br>**≤ 7 DOL**	**V1**<br>**1 mo**	**V2**<br>**3 mo**
Written Informed Consent	X		
Infants and family history	X		
Inclusion and exclusion criteria	X		
Randomization	X		
Antropometry	X	X	X
Body Composition	X		X
GI Tolerance		X	X
Stools collection	X	X	X
Adverse events			

8. **Sample collection and measurement methods**

8.1 Anthropometry

Patients will be weighed naked; an electronic scale will be used, with an accuracy of +/- 0.1 g.

The length will be measured with a baby's statimeter (Harpenden, Holtail Ltd, UK) with accuracy of +/- 0.1cm. The head circumference will be measured at the fronto-occipital level, with a well-stretched non-elastic meter with accuracy of ± 0.1 cm.

The values of the anthropometric parameters will then be reported on the percentiles by weight, length and head circumference of the World Health Organization present in the CRF data collection form (ANNEX 1).

8.2 Body composition

Body composition measurement will be performed through PEA POD (Pea Pod Infant Body Composition System, Life Measurement, Inc., Concord, CA, USA): an innovative, safe, easy to use, non-invasive and highly accurate system to determine the body composition even in the premature newborn. This tool allows to evaluate the quality and quantity of growth with particular attention to the percentage of fat mass. PEA POD is a double chamber system (test chamber and reference chamber). The volume of the subject is calculated by evaluating the variations in volume of the air contained in the two chambers in isothermal and adiabatic conditions. When the subject is introduced into the test chamber, it causes a disturbance of the volume of air inside it. Since the volume of the test chamber is reduced by the volume of the subject, the pressure inside this chamber increases inversely proportional to the reduction in the volume of air inside it. This pressure increase, compared with the pressure present in the reference chamber, allows to calculate the volume of the subject. The relationship between the weight and the volume of the subject allows to calculate the body density and, from this, the quantity of lean mass and fat mass. In order to obtain a valid measurement it is necessary for the child to lie in the naked test chamber for two

minutes. PEA-POD provides accurate values even when the infant is agitated or cries. PEA-POD allows assessing the body composition of children weighing less than 7 kg.

8.3 Collection of stool samples

The stools will be collected at the study points provided always at the same time of the day (morning) and stored in at least three aliquots per sample that will be stored in the freezer at -80 C. An aliquot will be used for the analysis of antimicrobial peptides (defensins, catelicidins) and for the measurement of IgA, one for microbiota analysis and the third for metabolome analysis. Measurements of antimicrobial peptides and IgA will be conducted by ELISA. The microbiota analysis will be conducted as a metagenomic analysis by sequencing hypervariable regions of the 16S rRNA genes, which are genetic signs attributable to different bacterial species (barcodes) using an ultra-deep sequencing technique using the MiSeq platform (Illumina), after having generated libraries using amplicon strategy that allow to obtain a faithful and comprehensive overview of the taxonomic content of the samples under analysis. Transcriptomic analyzes will also be performed to evaluate the transcriptional status of bacteria by generating RNA libraries to be analyzed by RNA sequencing technique.

The analyses will be carried out at the Department of Experimental Oncology of the European Institute of Milan, Italy and the Department of Translational Medical Sciences-Section of Pediatrics, University of Naples "Federico II" - Naples. Metabolomic analysis will also be performed on the stool samples collected. Metabolomics is the youngest of the "omics" technologies, aimed at identifying, quantifying and simultaneously characterizing thousands of low molecular weight metabolites (oligopeptides, AA, sugars, organic acids, bile acids, simple fatty acids, lipids, steroids, vitamins) present in cells, tissues, organs and biological fluids, which vary according to physiological stimuli, to the development and to the pathological state of a cell, tissue, organ or whole organism. Its components (metabolites) can be seen as the end product of gene expression or protein activity (enzymes), which thus define the biochemical phenotype of a biological system as a whole, including man. Metabolomics studies the alterations of the metabolic functions of the

systems by spectroscopic techniques, Nuclear Magnetic Resonance, and is capable of generating an instantaneous photo of the metabolic state of the biological system under examination. All costs will be covered by the sponsor.

8.4 Tolerance evaluation

The evaluation of the gastro-enteric tolerance of the formula will take place through a diary for the collection of data relating to tolerance (ANNEX 2), delivered to the parents of the participants at the time of enrollment and by means of a questionnaire inserted in the CRF (ANNEX 3). The parameters of gastrointestinal tolerance have been defined as indicated below:

Regurgitation: emission of small quantities of milk between meals

Vomiting: emission of milk after retching

Colic: intermittent attacks of abdominal pain when the infant cries and lifts the legs towards the abdomen with well-being between the episodes

Stools consistency: liquid: Figure 1; soft: Figure 2; formed: Figure 3; hard

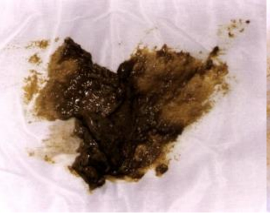
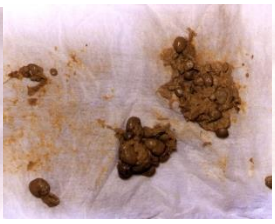
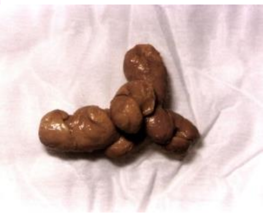

Figura 1 **Figura 2** **Figura 3**

8.5 Patient record

The data relating to the recruited subjects will be adequately recorded in the CRF patient card (ANNEX 3).

8.6 Concomitant therapies

The Investigator will record in the CRF all the drugs taken by the infant during the CRF study (ANNEX 3).

9. Product description

9.1 Formulas composition

The composition of the formulas is shown in ANNEX 4

9.2 Dosages

The two formulas will be in powder formulation. Each pack will contain 800 g of powdered milk. The concentration, for all the formulas, will be obtained by diluting one scoop of powder for every 30 cc of water (minimally mineralized or previously boiled). The volume of milk required will be defined on the basis of the correct weight and age of the infant; the quantity assumed will be reported in the patient file. During each visit, the experimenter will also provide the parents with the formula-fed infants, together with the product, instructions for its preparation and storage. Infants will be fed with the formulas assigned to them until the end of the study.

9.3 Quality control

The quality control of the products will be carried out by Heinz Italia S.p.A., in compliance with the European regulations in force, at the time of production. The same regulation will be applied for the storage, transport and distribution of the product. The lot, the identification code and the expiry date will be reported on each package.

9.4 Packaging and labeling

The formulas for infants will be packaged and labeled by Heinz Italia S.p.A.

9.5 Distribution and storage of the product

The amount of milk formulated necessary for the study will be provided by Heinz Italia S.p.A. free of charge and will be stored in a dry environment at room temperature.

The supply of the formula assigned to each enlisted person will start after the approval of the study by the Ethics Committee.

For the first 3 months of life, infants will receive study formulas free of charge based on randomization. The sponsor also undertakes to provide free milk formulated to all children even at the end of the study until the introduction of complementary nutrition, which will be decided exclusively by the family pediatrician.

10. Adverse events

All the adverse events observed during the study will be recorded using a questionnaire. Particular attention will be given to the following adverse events: diarrhea, crying, gas colic, regurgitation, vomiting, cutaneous manifestations, fever. Data on severity, duration, possible relationship with the formula, outcome and action taken, of the events (diarrhea, crying, gas colic, regurgitation, vomiting, cutaneous manifestations, fever) that required the intervention of the pediatrician doctor and of all those not listed above, even if they did not request the intervention of the pediatrician (ANNEX 5).

Adverse Event Definition (AE)

An AE is any adverse medical or other incident occurring in a subject participating in a clinical trial to which a product has been administered and which does not necessarily have to have a causal relationship with such treatment. An adverse event can therefore be any sign, unfavorable or unwanted, a symptom or an illness or other unfavourable event occurring in coincidence with the use of the product, whether associated or not with the product.

Serious Adverse Event Definition (SAE)

An SAE is any unfavorable clinical manifestation (whether or not associated with the product being studied) that is at any dosage:

• Fatal

• Life threatening: the subject risked dying when the event occurred.

• Request hospitalization or extension. Admission to hospital and / or surgical procedures planned before or during the study are not considered adverse events if the disease existed prior to the subject's enrollment in the study, if it did not deteriorate during the study

• Persistent or significant incapacity / disabilities

• Both a congenital anomaly / a birth defect

• Other: significant medical events that may damage the subject and suggest precautions or require preventive treatment so that the conditions listed above do not occur.

10.1 Reporting of serious adverse events

In the case of an SAE, the investigator must complete, sign and date the SAE reporting form, check the correctness and consistency of the data and send a copy via fax (within 24 hours of the event being detected) to:

Dr. Andrea Budelli Tel 347 4763379 FAX 02 5256 2929 Email: andrea.budelli@nl.hjheinz.com

10.2 Follow-up of adverse events

Any AEs observed since randomization until the end of the study should be followed up to its resolution. With resolution it is understood that the infant returns to the state of basic health or that the experimenter does not wait for a worsening of the adverse event.

10.3 Post-study serious adverse events All SAEs that occur within 30 days of the last product administration must be communicated as specified in paragraph 10.1.

10.4 Definition of the relationship

The relationship of adverse events with the product under study will be evaluated using the following definitions:

• Existing relationship: an adverse event commonly associated with the administration of the formula. A temporary relationship with the administration of the clinical product, with no apparent evidence of another etiology.

• Non-existing relationship: an adverse effect usually not associated with the administration of the formula. No temporary relationship with the administration of the clinical product and possibility of further etiology.

• Unknown report: there is insufficient data to evaluate the relationship of the adverse event both with the administered clinical product and with any study procedure.

10.5 Responsibility for reporting serious adverse events

In the case of a Serious Adverse Event considered not associated with the administration of the product being studied, the investigator must report the event as well as the Sponsor also to the competent Ethics Committee, if specifically requested by the regulation of the same. If you have a Serious Adverse Event where you don't can exclude the association with the administration of the product in study, the experimenter will have to signal the event beyond that to the Sponsor also to the Ethical Committee, if unexpected event, and to the Health Direction where required.

9. Statistical Analysis

11.1 Calculation of sample size

The sample size was determined to identify between the two groups of infants, the one who received the fermented formulated milk and the one who received a standard formulated milk (Plasmon Primigiorni), a difference in the content of faecal α-defensins on samples of feces. Given the results concerning the effect of a treatment with fermented milk with L. paracasei CBA L74 on faecal α-defensins in children attending preschool centers average value (DS) vs placebo [4.8 (4.2) vs 1 (0.6), respectively] we have calculated that 12 babies are needed per group with a power of 84% and an alpha of 0.05. Assuming a 30% dropout rate, at least 16 newborns per group must be recruited.

11.2 Criteria for the evaluation of variables

Descriptive analyses will be carried out by calculating the mean, median and standard deviations for continuous variables and expressing the distribution of frequencies for discrete variables. The main analysis will be based on tests for independent samples, Student's or Mann-Whitney's tests depending on the distribution of the outcome variables. To jointly analyse the trend over time from V0 to V2 of the outcome variables in the two groups, the one that received the milk added with a fermented product and the one that received a standard milk, based on the type of delivery (spontaneous or caesarean section) regression models will be used for repeated measures (random effects models or GEE models).

12. Legal and ethical requirements

12.1 Legality of the study

The study will be conducted in accordance with current legal requirements.

12.2 Protection of patient data privacy

The personal data of the participants and the results obtained will be kept confidential. The parents of the newborns participating in the study will receive the information and consent to the processing of personal data of minors (ANNEX 6).

12.3 Informed consent

The study will be described and explained in detail to the parents of each new-born baby considered eligible by one of the doctors in charge. A copy of the informed consent will be given, which must be dated and signed by parents and by the doctor in charge. No patient will be enrolled without duly signed informed consent. (ANNEX 7).

12.4 Paediatrician Care Information At the time of enrollment, each newborn's parents will receive an information letter from the study for the Paediatrician (ANNEX 8).

12.5 Approval by the ethics committee

The study will be submitted for approval by the Ethics Committee. The study will only start after authorization.

12.6 Quality Assurance and Quality Control

In accordance with the Good Clinical Practice Guidelines (CPMP / ICH / 135/95), the Sponsor is responsible for the quality of the study data. The data generated by this clinical study will be analysed in accordance with the procedures established by the Statistical Unit of the Center of Milan.

13. Responsibility

13.1 Responsibilities of the investigators

Investigators will be responsible for all clinical and assessment procedures that infants will undergo during the study and as required by this protocol.

13.2 Responsibilities of the sponsor

Heinz Italia S.p.A., as Sponsor, is responsible for the quality of the formulas under study.

14. Documentation and access to data

14.1 Documentation of the participation of the subjects in the study

The experimenter must report, in an Infant Identification List, the identification data (initial of the first and last name, date of birth and identification code) of each infant for which the consent of the parents or the parent has been obtained legal guardian (s). This must also be done in case the infant has not taken the product being tested. The investigator will have to keep this list for a period of at least 10 years after the end of the study within the study archive.

14.2 Documentation of essential documents

The investigator / institution is responsible for keeping the study documents as specified in the Good Clinical Practice Guidelines (CPMP / ICH / 135/95) and as required by the applicable regulatory requirements. The investigator / institution must prevent the accidental destruction of these documents.

14.3 Data protection

To safeguard the identity of the infant, an identification code will be assigned to it which must be used by the investigator instead of the name of the infant. To safeguard the identity of the infant, each infant will be identified throughout the duration of the study by the initials (the first letter of the name followed by the first letter of the surname) and by an alphanumeric identification code corresponding to the progressive number assigned at the time of enrollment by the experimenter.

This number must be used on all documents and must have a one-to-one correspondence with the date of birth.

Personal information will be treated as confidential, but it will need to be reviewed by authorized representatives of Heinz Italia S.p.A (monitor and auditor), or by the companies delegated to conduct the study, the Ethics Committee and the regulatory authorities. The consent of the parents or of the legal guardian (s) of the infant to the direct access of the data of his infant must be obtained before participating in the study.

14.4 Documents of study participants

The investigator will have to allow the employees authorized by the Sponsor to conduct the study and the regulatory agencies, to enter and inspect every place where the product and documents on the product will be stored and to inspect and copy all the documents, including those of infants.

The Data Collection Forms (CRFs) must be available for review by the Sponsor, the Clinical Monitors responsible for conducting the study and regulatory agencies.

15. Administrative procedures

15.1 Amendments to the protocol

The modifications of the signed protocol will be possible only through the drafting of amendments to the approved protocol and with the agreement of all responsible persons. The procedure for the approval of an amendment to the protocol is identical to that for the approval of the protocol itself. The Ethics Committee must be informed of all amendments to the protocol and its opinion and the re-evaluation of the ethical aspects of the study must be requested. The Investigator will not be able to implement any deviation or change from the protocol without the agreement of the Sponsor and before the review and approval / documented favorable opinion of the Ethics Committee, except when it will be necessary to eliminate an immediate risk to the infants of the study, or if the changes will involve aspects only logistic or administrative (for example, change of phone numbers or

contacts). The amendments to the protocol will be submitted to the competent authorities as requested by the appropriate competent authorities.

15.2 Disclosure of information and results

With the signing of the protocol, each participating investigator agrees to maintain confidentiality regarding all information and results concerning the study and the product under study until the data / results are published. The obligation of confidence applies to all personnel involved in the experimental centers. The publication of the study results requires a mutual agreement between investigators and Heinz Italia S.p.A. The possible denial to the publication by the experimenters or by Heinz Italia Sp A. must be adequately motivated and must not be unreasonably denied.

15.3 Premature termination of the study

Sponsor may terminate the study in an experimental center in particular for one of the following reasons:

- The center cannot include an adequate number of infants
- Serious and / or persistent non-compliance with the protocol
- Negligence or premeditated false documentation in CRFs
- Inadequate cooperation with the Sponsor, or its representatives
- Non-compliance with the requirements of Good Clinical Practice and regulators
- The experimenter requested the interruption.

If the study terminated prematurely or was suspended for any reason, the parents of the infants, parents or legal guardian / s should be promptly informed. Appropriate treatment and follow-up should be provided to infants and, where required by the applicable regulatory requirements, the relevant regulatory authorities will be informed. The Ethics Committees will be promptly informed and a detailed written explanation of the interruption will be provided.

15.4 Insurance for study participants

In the event of serious adverse events to the child directly or indirectly resulting from the study, the sponsor has stipulated a third party liability insurance policy with the Allianz Company and RCO (ATTACHMENT 9), to cover the risks for any damage deriving from carrying out the Project with a maximum per protocol of Euro 7,500,000.00 (seven million five hundred thousand / 00) and per subject of Euro 1,000,000.00 (one million / 00) starting from 00 on 01/05/2015 and valid until 24 hours of 04/30/2016. If the insurance certificate does not cover the entire duration of the trial, the sponsor will renew the insurance coverage by its expiry date.

Subsequent coverage: the coverage is considered valid and effective for damage caused by facts during the period of validity of this contract, provided that such damages occurred no later than 120 months from the end of the trial and / or this policy, and for them both a claim for compensation was submitted no later than 120 months from the end of the trial itself. By "from the end of the experiment" we mean what is established by the D.M. 14.07.09 to the Art. 1.3.

15.5 Registration of the protocol

In accordance with the guidelines and recommendations specified by The International Committee of Medical Journal Editors (ICMJE) the study will be registered at http://www.clinicaltrials.gov.

16. References

-Palmer C, Bik EM, DiGiulio DB, Relman DA, Brown PO (2007) Development of the human infant intestinal microbiota. PLoS Biology 5: e177.
- Madan JC, Farzan SF, Hibberd PL, Karagas MR (2012) Normal neonatal microbiome variation in relation to environmental factors, infection and allergy. Current opinion in pediatrics 24: 753–759.
-Maynard CL, Elson CO, Hatton RD, Weaver CT (2012) Reciprocal interactions of the intestinal microbiota and immune system. Nature 489:231–241.

-Hooper LV, Littman DR, Macpherson AJ (2012) Interactions between the microbiota and the immune system. Science 336: 1268–1273.

-Agostoni C, Goulet O, Kolacek S, Koletzko B, Moreno L, Puntis J, Rigo J, Shamir R, Szajewska H, Turck D; ESPGHAN Committee on Nutrition (2007) Fermented infant formulae without live bacteria. Journal of pediatric gastroenterology and nutrition 44:392-397.

-Sim K, Powell E, Shaw AG, McClure Z, Bangham M, et al. (2013) The neonatal gastrointestinal microbiota: the foundation of future health? Archives of disease in childhood Fetal and neonatal edition 98: F362–364.

-Agostoni C, Braegger C, Decsi T, Kolacek S, Koletzko B, et al. (2009) Breastfeeding: A commentary by the ESPGHAN Committee on Nutrition. Journal of pediatric gastroenterology and nutrition 49: 112–125.

- Harmsen HJ, Wildeboer-Veloo AC, Raangs GC, Wagendorp AA, Klijn N, et al. (2000) Analysis of intestinal flora development in breast-fed and formula-fed infants by using molecular identification and detection methods. Journal of pediatric gastroenterology and nutrition 30: 61–67.

- Howie PW, Forsyth JS, Ogston SA, Clark A, Florey CD (1990) Protective effect of breast feeding against infection. Bmj 300: 11–16.

- Hasselbalch H, Jeppesen DL, Engelmann MD, Michaelsen KF, Nielsen MB (1996) Decreased thymus size in formula-fed infants compared with breastfed infants. Actapaediatrica 85: 1029–1032.

- Koletzko B, Baker S, Cleghorn G, Neto UF, Gopalan S, et al. (2005) Global standard for the composition of infant formula: recommendations of an ESPGHAN coordinated international expert group. Journal of pediatric gastroenterology and nutrition 41: 584–599.

-Azad MB, Konya T, Maughan H, Guttman DS, Field CJ, Cet al. (2013) Gut microbiota of healthy Canadian infants: profiles by mode of delivery and infant diet at 4 months. CMAJ 185: 385-394.

-Riva E, Verduci E, Agostoni C, Giovannini M (2005) Closer to the goldstandard: an appraisal of formulae available in Italy for use in formula-fedinfants. The Journal of international medical research 33: 595–611.

-Mullie C, Yazourh A, Thibault H, Odou MF, Singer E, et al. (2004) Increased poliovirus-specific intestinal antibody response coincides with promotion of Bifidobacteriumlongum-infantis and

Bifidobacteriumbreve in infants: a randomized, double-blind, placebo-controlled trial. Pediatric research 56: 791–795.

- Agostoni C, Goulet O, Kolacek S, Koletzko B, Moreno L, et al. (2007) Fermented infant formulae without live bacteria. J PediatrGastroenterolNutr 44:392-397.

-Indrio F, Ladisa G, Mautone A, Montagna O (2007) Effect of a fermented formula on thymus size and stool pH in healthy term infants. Pediatric research 62: 98–100.

-Thibault H, Aubert-Jacquin C, Goulet O (2004) Effects of long-term consumption of a fermented infant formula (with Bifidobacteriumbreve c50 and Streptococcus thermophilus 065) on acute diarrhea in healthy infants. Journal of pediatric gastroenterology and nutrition 39: 147–152.

-Nocerino R, Paparo L, Maddalena Y, Amoroso A, Pezzella V et al. Cow's milk and rice fermented with Lactobacillus paracasei CBA L74 prevent gastrointestinal and respiratory tract infections in young children: a prospective randomized study (2014). J Pediatr Gastroenterol Nutr 58: 445.

-Zagato E, Mileti E, Massimiliano L, Fasano F, Budelli A, et al. Lactobacillus paracasei CBA L74 metabolic products and fermented milk for infant formula have anti-inflammatory activity on dendritic cells in vitro and protective effects against colitis and an enteric pathogen in vivo (2014) PLoS One 9: e87615.

-The EFSA Panel on Additives and Products or Substances used in Animal Feed Update of the criteria used in the assessment of bacterial resistance to antibiotics of human or veterinary importance (2008) The EFSA Journal 732: 1-15.

-The EFSA Panel on Biological Hazards The maintenance of the list of QPS microorganisms intentionally added to food or feed (2008) The EFSA Journal 923: 1-48.
