# Supplementary material for: Analysis of immune, microbiota and metabolome maturation in infants in a clinical trial of Lactobacillus paracasei CBA L74-fermented formula
Source: Nat Commun. 2020 Jun 1;11:2703. doi: 10.1038/s41467-020-16582-1 (PMC7264213; doi:10.1038/s41467-020-16582-1)
Supplement: Supplementary file 5 — Reporting Summary [file 41467_2020_16582_MOESM5_ESM.pdf]

nature research

Corresponding author(s): MARIA RESCIGNO and PAOLA ROGGERO

Last updated by author(s): Apr 9, 2020

Reporting Summary

Nature Research wishes to improve the reproducibility of the work that we publish. This form provides structure for consistency and transparency in reporting. For further information on Nature Research policies, see Authors & Referees and the Editorial Policy Checklist .

Statistics

For all statistical analyses, confirm that the following items are present in the figure legend, table legend, main text, or Methods section.

n/a	Confirmed	
☐	☒	The exact sample size (*n*) for each experimental group/condition, given as a discrete number and unit of measurement
☐	☒	A statement on whether measurements were taken from distinct samples or whether the same sample was measured repeatedly
☐	☒	The statistical test(s) used AND whether they are one- or two-sided<br>*Only common tests should be described solely by name; describe more complex techniques in the Methods section.*
☐	☒	A description of all covariates tested
☐	☒	A description of any assumptions or corrections, such as tests of normality and adjustment for multiple comparisons
☐	☒	A full description of the statistical parameters including central tendency (e.g. means) or other basic estimates (e.g. regression coefficient) AND variation (e.g. standard deviation) or associated estimates of uncertainty (e.g. confidence intervals)
☒	☐	For null hypothesis testing, the test statistic (e.g. *F*, *t*, *r*) with confidence intervals, effect sizes, degrees of freedom and *P* value noted<br>*Give P values as exact values whenever suitable.*
☒	☐	For Bayesian analysis, information on the choice of priors and Markov chain Monte Carlo settings
☒	☐	For hierarchical and complex designs, identification of the appropriate level for tests and full reporting of outcomes
☒	☐	Estimates of effect sizes (e.g. Cohen's *d*, Pearson's *r*), indicating how they were calculated

Our web collection on statistics for biologists contains articles on many of the points above.

Software and code

Policy information about availability of computer code

Data collection	Weight was measured on an electronic scale accurate to 0.1 g (PEA POD Infant Body Composition System; Cosmed, Italy)<br>Body length was measured to the nearest 1 mm on a Harpenden neonatometer (Holtain, Crymych, UK).<br>Body composition was assessed at enrollment and at three months using an air-displacement plethysmography (PEA POD Infant Body Composition System; COSMED, Italy). ELISA plate reader (Biocompare, San Francisco, CA, USA), Illumina MiSeq System (Illumina, San Diego), GC-2010 Plus gas chromatograph coupled to a 2010 Plus single quadrupole mass spectrometer (Shimadzu Corp., Kyoto, Japan), SolariX XR 7T (Bruker Daltonics).
Data analysis	Qiime2-2019.4, R 3.5.1, ggplot2 3.1.0, lmerTest 3.1-0, emmeans 1.4, psych 1.8.12, Prism (GraphPad software, v6.01b), Compass Data Analysis version 4.2, Compound Crawler ver. 3.0, SPSS v12.

For manuscripts utilizing custom algorithms or software that are central to the research but not yet described in published literature, software must be made available to editors/reviewers. We strongly encourage code deposition in a community repository (e.g. GitHub). See the Nature Research guidelines for submitting code & software for further information.

Data

Policy information about availability of data

All manuscripts must include a data availability statement. This statement should provide the following information, where applicable:

- Accession codes, unique identifiers, or web links for publicly available datasets
- A list of figures that have associated raw data
- A description of any restrictions on data availability

Data supporting the findings of this study are available within the paper and its Supplementary Information files. Data underlying Figs 2, 3a, 3c, Supp Figs 2-5 are provided as Source Data files. All other data are available from the corresponding author upon reasonable requests. The datasets generated and analysed during the current study regarding the 16SrRNA microbiota analysis are available in the Sequence Read Archive repository (SRA accession: PRJNA608934); those regarding the metabolome analysis are identified in MetaboLights as MTBLS1532 [https://www.ebi.ac.uk/metabolights/MTBLS1532].

Field-specific reporting

Please select the one below that is the best fit for your research. If you are not sure, read the appropriate sections before making your selection.

☒ Life sciences ☐ Behavioural & social sciences ☐ Ecological, evolutionary & environmental sciences

For a reference copy of the document with all sections, see nature.com/documents/nr-reporting-summary-flat.pdf

Life sciences study design

All studies must disclose on these points even when the disclosure is negative.

Sample size	The power of the study was calculated based on our previous clinical trials to evaluate the effect of a treatment with milk fermented with L. paracasei CBA L74 in young children attending preschool centres, on different biological parameters (sIgA, defensins) as well as clinical outcome in preventing seasonal infectious diseases 31,32. The primary objective of the current clinical trial was to evaluate the increase of sIgA in response to the different diets. Based on this parameter, the power of this study was 80% and an alpha of 0.05, therefore we calculated to enroll, after drop out, 13 newborns per each group .<br>Given the very young age of babies at enrollment, we could not collect the feces from all of the newborns and hence the number of samples has been reduced to 54 (9 infants per group) to ensure a consistent number throughout the study groups.
Data exclusions	No data were excluded from the analysis
Replication	This was a clinical trial and there were no replicates
Randomization	The randomization schedule was computer-generated and stratified on type of delivery using 2 computer-generated randomization lists, 1 for vaginal delivered infants and 1 for cesarean section delivered infants.
Blinding	Yes. It was a double-blind clinical trial

Reporting for specific materials, systems and methods

We require information from authors about some types of materials, experimental systems and methods used in many studies. Here, indicate whether each material, system or method listed is relevant to your study. If you are not sure if a list item applies to your research, read the appropriate section before selecting a response.

Materials & experimental systems

n/a	Involved in the study	
☐	☒	Antibodies
☒	☐	Eukaryotic cell lines
☒	☐	Palaeontology
☒	☐	Animals and other organisms
☐	☒	Human research participants
☐	☒	Clinical data

Methods

n/a	Involved in the study	
☒	☐	ChIP-seq
☒	☐	Flow cytometry
☒	☐	MRI-based neuroimaging

Antibodies

Antibodies used	HNP 1-3 was measured by ELISA using a human kit (Hycult biotechnology, Uden, The Netherlands); HBD-2 by ELISA using a human kit (Phoenix Pharmaceuticals, Inc., Burlingame, CA, USA); LL-37 by ELISA using an human kit (Hycult biotechnology, Uden, The Netherlands), and sIgA by indirect enzyme immunoassay (Salimetrics LLC, Carlsbad, CA, USA).
Validation	These were all validated kits

Human research participants

Policy information about studies involving human research participants

Population characteristics	Healthy full term infants were screened for participation in the study. Inclusion criteria were: singleton, full-term infants (gestational age from 37/0 to 41/6 weeks), with a birth weight adequate for gestational age (>10th percentile and <90th percentile for gestational age) according to the World Health Organization growth charts (available at http://www.who.int/childgrowth/standards/en/), when entering the study. Exclusion criteria were the presence of congenital diseases, chromosomal abnormalities and/or conditions that could interfere with growth, such as brain, metabolic, cardiac and gastrointestinal diseases, perinatal infections, being born to a mother affected by endocrine and/or metabolic diseases, or having a family history of allergic disease. The mean weight and gestational age at birth of infants enrolled were 3243±372 g and 38.5±1.0 weeks. The age range at enrolment was 0-7 days (3.3±1.6 days). Therefore, as the mean weight and gestational age at birth were similar among groups, we did not perform any correction for further analyses.
Recruitment	All the mothers of infants evaluated for the study were encouraged to breastfed their infants for at least 4 months: if they could not or intended not to breastfeed their infants, the study investigators asked them for their consent to participate in the study within 7 days after birth. Newborns were randomized to receive until the third month of age standard formula containing 2.3 g/100 g of cow's milk powder fermented with the probiotic L. paracasei CBA L74 (formula F group) or standard formula (formula S group). The reference group was constituted only by breastfed infants and not by mother milk bottle-fed infants. It is a double-blind randomized trial which minimize the introduction of biases.
Ethics oversight	The study was approved by the Ethics Committee and conducted in accordance with Good Clinical Practice and the principles and rules of Declaration of Helsinki. Parents or legal caregivers provided written informed consent before the enrolment of their infants in the study. The trial was registered in the Clinical Trials Protocol Registration System (ClinicalTrials.gov) with the identifier NCT03637894.

Note that full information on the approval of the study protocol must also be provided in the manuscript.

Clinical data

Policy information about clinical studies

All manuscripts should comply with the ICMJEguidelines for publication of clinical research and a completedCONSORT checklist must be included with all submissions.

Clinical trial registration	Clinical Trials Protocol Registration System (ClinicalTrials.gov) with the identifier NCT03637894
Study protocol	All the mothers of infants evaluated for the study were encouraged to breastfed their infants for at least 4 months: if they could not or intended not to breastfeed their infants, the study investigators asked them for their consent to participate in the study within 7 days after birth. Newborns were randomized to receive until the third month of age standard formula containing 2.3 g/100 g of cow's milk powder fermented with the probiotic L. paracasei CBA L74 (formula F group) or standard formula (formula S group). The reference group was constituted only by breastfed infants and not by mother milk bottle-fed infants.<br>The composition of the study dietary products has already been described in 31. The powder was provided by Heinz Italia SpA, Latina, Italy, an affiliate of H.J. Heinz Company, Pittsburgh, PA, USA. The fermented milk was prepared from skimmed milk fermented with L. paracasei CBA L74, which was isolated from the feces of healthy infants. The fermentation was initiated with 106 bacteria, and stopped when reaching 5.9 x 109 colony-forming units/g (after a 15 h incubation at 37 °C). Live bacteria were then inactivated by heating at 85 °C for 20 s and the formula was spray-dried. Study products were provided already prepared in tins containing 400 g of powder and the packaging was similar between S and F formulas. Study products were stored at room temperature and in a dry environment.<br>The randomization schedule was computer-generated and stratified on type of delivery using 2 computer-generated randomization lists, 1 for vaginal delivered infants and 1 for cesarean section delivered infants. Study formulas were formulated into powder and were reconstituted at 13.3% and the packages were the same and identified with letters to make them unrecognizable. They had similar energy and macronutrient contents but they differed by the presence of the fermented product. Specifically the formula F content was: energy 69 kcal/100 ml, fats: 3.6 g/100 ml, proteins: 1.4 g/100 ml, carbohydrates: 7.4 g/100 ml; formula S content was: energy 68 kcal/100 ml, fats: 3.6 g/100 ml, proteins: 1.4 g/100 ml, carbohydrates: 7.3 g/100 ml. Infants fed with exclusive breast milk for the first three months of life represented the reference group.<br>All the mothers of infants evaluated for the study were encouraged to breastfed their infants for at least 4 months: if they could not or intended not to breastfeed their infants, the study investigators asked them for their consent to participate in the study within 7 days after birth. Newborns were randomized to receive until the third month of age standard formula containing 2.3 g/100 g of cow's milk powder fermented with the probiotic L. paracasei CBA L74 (formula F group) or standard formula (formula S group). The reference group was constituted only by breastfed infants and not by mother milk bottle-fed infants.<br>The composition of the study dietary products has already been described in 31. The powder was provided by Heinz Italia SpA, Latina, Italy, an affiliate of H.J. Heinz Company, Pittsburgh, PA, USA. The fermented milk was prepared from skimmed milk fermented with L. paracasei CBA L74, which was isolated from the feces of healthy infants. The fermentation was initiated with 106 bacteria, and stopped when reaching 5.9 x 109 colony-forming units/g (after a 15 h incubation at 37 °C). Live bacteria were then inactivated by heating at 85 °C for 20 s and the formula was spray-dried. Study products were provided already prepared in tins containing 400 g of powder and the packaging was similar between S and F formulas. Study products were stored at room temperature and in a dry environment.<br>The randomization schedule was computer-generated and stratified on type of delivery using 2 computer-generated randomization lists, 1 for vaginal delivered infants and 1 for cesarean section delivered infants. Study formulas were formulated into powder and were reconstituted at 13.3% and the packages were the same and identified with letters to make them unrecognizable. They had similar energy and macronutrient contents but they differed by the presence of the fermented product. Specifically the formula F content was: energy 69 kcal/100 ml, fats: 3.6 g/100 ml, proteins: 1.4 g/100 ml, carbohydrates: 7.4 g/100 ml; formula S content was: energy 68 kcal/100 ml, fats: 3.6 g/100 ml, proteins: 1.4 g/100 ml, carbohydrates: 7.3 g/100

ml. Infants fed with exclusive breast milk for the first three months of life represented the reference group.

Data collection

Anthropometric and body composition measurements were performed by the same medical investigator who was blinded to the allocated treatment. Body weight, length and head circumference were measured at birth, one and three months according to standard procedures 48,49. Weight was measured on an electronic scale accurate to 0.1 g (PEA POD Infant Body Composition System; Cosmed, Italy). Body length was measured to the nearest 1 mm on a Harpenden neonatometer (Holtain, Crymych, UK). Head circumference was measured to the nearest 1 mm using non-stretch measuring tape. Body composition was assessed at enrollment and at three months using an air-displacement plethysmography (PEA POD Infant Body Composition System; COSMED, Italy). A detailed description of the PEA POD's physical design, operating principles, validation, and measurement procedures is provided elsewhere 50-52.

All parents were instructed to fill a clinical diary. Specifically they had to record any use of drugs, possible hospitalizations; possible adverse events; consumption of the study products and data regarding gastrointestinal tolerance. The following indicators of tolerability were collected through multiple-choice questions: volume of formula intake, daily frequency of stool passage, episodes and type of vomit or spitting, episodes of flatulence and abdominal pain (infantile colics: defined as intermittent attacks of abdominal pain when the baby screams and draws up his/her legs but is well between episodes).

Infant colics were further classified as severe if the episodes occurred more than twice a day. The diary served as an indicator for the need of a medical examination and as a consistent way of recording and recalling symptoms. Parents were instructed to contact the investigator if necessary and to avoid the use of prebiotics, probiotics, symbiotics, and immune stimulating products during the 3-month study period.

Outcomes

The power of the study was calculated based on our previous clinical trials to evaluate the effect of a treatment with milk fermented with L. paracasei CBA L74 in young children attending preschool centres, on different biological parameters (sIgA, defensins) as well as clinical outcome in preventing seasonal infectious diseases 31,32. The primary objective of the current clinical trial was to evaluate the increase of sIgA and anti-microbial peptides such as catelecidins, alpha and beta defensins in response to the different diets. Based on this parameter, the power of this study was 80% and an alpha of 0.05, therefore we calculated to enroll, after drop out, 13 newborns per each group.

The secondary outcomes were:

- To evaluate the tolerance in the two groups of infants fed with the two formulas compared to breastfed infants;
- To evaluate the modifications of the intestinal microbiota in the two groups of infants fed with the two formulas compared to breastfed infants.
